# Thioredoxin and glutathione systems differ in parasitic and free-living platyhelminths

**DOI:** 10.1186/1471-2164-11-237

**Published:** 2010-04-13

**Authors:** Lucía Otero, Mariana Bonilla, Anna V Protasio, Cecilia Fernández, Vadim N Gladyshev, Gustavo Salinas

**Affiliations:** 1 Cátedra de Inmunología, Facultad de Química, Instituto de Higiene, Universidad de la República, Avda. A. Navarro 3051, Montevideo, Uruguay; 2 Wellcome Trust Sanger Institute, Wellcome Trust Genome Campus, Hinxton CB10 1SA, UK; 3 Brigham and Women's Hospital, Harvard Medical School, Boston, MA 02115, USA

## Abstract

**Background:**

The thioredoxin and/or glutathione pathways occur in all organisms. They provide electrons for deoxyribonucleotide synthesis, function as antioxidant defenses, in detoxification, Fe/S biogenesis and participate in a variety of cellular processes. In contrast to their mammalian hosts, platyhelminth (flatworm) parasites studied so far, lack conventional thioredoxin and glutathione systems. Instead, they possess a linked thioredoxin-glutathione system with the selenocysteine-containing enzyme thioredoxin glutathione reductase (TGR) as the single redox hub that controls the overall redox homeostasis. TGR has been recently validated as a drug target for schistosomiasis and new drug leads targeting TGR have recently been identified for these platyhelminth infections that affect more than 200 million people and for which a single drug is currently available. Little is known regarding the genomic structure of flatworm TGRs, the expression of TGR variants and whether the absence of conventional thioredoxin and glutathione systems is a signature of the entire platyhelminth phylum.

**Results:**

We examine platyhelminth genomes and transcriptomes and find that all platyhelminth parasites (from classes Cestoda and Trematoda) conform to a biochemical scenario involving, exclusively, a selenium-dependent linked thioredoxin-glutathione system having TGR as a central redox hub. In contrast, the free-living platyhelminth *Schmidtea mediterranea *(Class Turbellaria) possesses conventional and linked thioredoxin and glutathione systems. We identify TGR variants in *Schistosoma *spp. derived from a single gene, and demonstrate their expression. We also provide experimental evidence that alternative initiation of transcription and alternative transcript processing contribute to the generation of TGR variants in platyhelminth parasites.

**Conclusions:**

Our results indicate that thioredoxin and glutathione pathways differ in parasitic and free-living flatworms and that canonical enzymes were specifically lost in the parasitic lineage. Platyhelminth parasites possess a unique and simplified redox system for diverse essential processes, and thus TGR is an excellent drug target for platyhelminth infections. Inhibition of the central redox wire hub would lead to overall disruption of redox homeostasis and disable DNA synthesis.

## Background

Platyhelminths (commonly known as flatworms) are a metazoan phylum that includes the neodermata lineage, composed exclusively of parasitic taxa, and the turbellaria lineage, mostly composed of free-living taxa [1]. Neodermatan flatworms comprise the classes Cestoda, Trematoda and Monogenea, of which the first two include the agents of serious human diseases. Notably, trematode infections caused by *Schistosoma *spp. affect more than 200 million people in Africa, South America and Asia [2]. Cestode infections are less prevalent, but include severe human diseases such as cysticercosis and hydatid disease, caused by *Taenia solium *and *Echinococcus *spp., respectively [3]. As yet, there is no vaccine that can prevent platyhelminth infections in humans, neither there are trials underway. Although chemotherapy is the mainstay of control, very few effective drugs are currently used to treat platyhelminth infections, being praziquantel the single drug readily available for schistosomiasis treatment [4]

In most organisms, including the mammalian hosts of platyhelminth parasites, cellular redox homeostasis, antioxidant defenses and supply of electrons for deoxyribonucleotide synthesis rely on two major and independent pathways: the glutathione (GSH) and the thioredoxin (Trx) systems [5], which have overlapping and differential targets, and function in a great variety of biological processes. These pathways operate through redox cascades that involve transfer of reducing equivalents from NADPH to targets through a series of dithiol-disulfide reactions or variations of this theme (*e.g*. when selenocysteine, Sec, replaces cysteine) [6]. The core enzymes of these pathways are glutathione reductase (GR) and thioredoxin reductase (TR), both of which are pyridine-nucleotide thiol-disulfide oxidoreductases that reduce the oxidized tripeptide glutathione (GSSG) and the oxidized disulfide reductase thioredoxin (Trx), respectively. In turn, GSH and Trx transfer electrons to downstream targets. Platyhelminth parasites, unlike their mammalian hosts, lack conventional GR and TR enzymes, and hence canonical thioredoxin and glutathione systems [7-9]. Instead, they possess a linked glutathione thioredoxin system that relies exclusively on the selenoenzyme thioredoxin glutathione reductase (TGR) for provision of reducing equivalents to both pathways. TGR achieves this broad substrate specificity by a fusion of an N-terminal glutaredoxin (Grx) domain to TR domains [10]. In the platyhelminth parasite *Echinococcus granulosus*, cytosolic and mitochondrial TGR variants derived from a single gene have been reported [11] and functional thioredoxin-glutathione systems have been recently described in both compartments [12], whereas only a cytosolic variant of TGR has been described in *S. mansoni *[13]. The differences in the thioredoxin and glutathione pathways between parasitic flatworms and their mammalian hosts, and the lack of redundancy of these redox pathways have prompted studies which have recently resulted in validation of TGR as a novel drug target for platyhelminths [14]. Indeed, new drug leads have been identified by quantitative high-throughput screenings using *Schistosoma mansoni *TGR as a target [15].

The fact that TGR is an essential enzyme that controls the overall redox homeostasis in these parasites warrants further studies on flatworm TGRs. In particular, little is known relating the genomic structure of flatworm TGRs, whether additional TGR variants are expressed, and how the variants are generated. More importantly, it is not known whether the presence of TGR and the absence of TR and GR genes is a signature of the platyhelminth lineage. In this work, we report that additional parasitic flatworms possess TGR and lack TR and GR. In contrast, the free-living platyhelminth *Schmidtea mediterranea *possesses TR, GR and TGR genes. In addition we investigate the existence, generation and expression of TGR variants in parasitic flatworms.

## Results

### Analysis of thioredoxin and glutathione systems in free-living and parasitic platyhelminths

**Figure 1:**
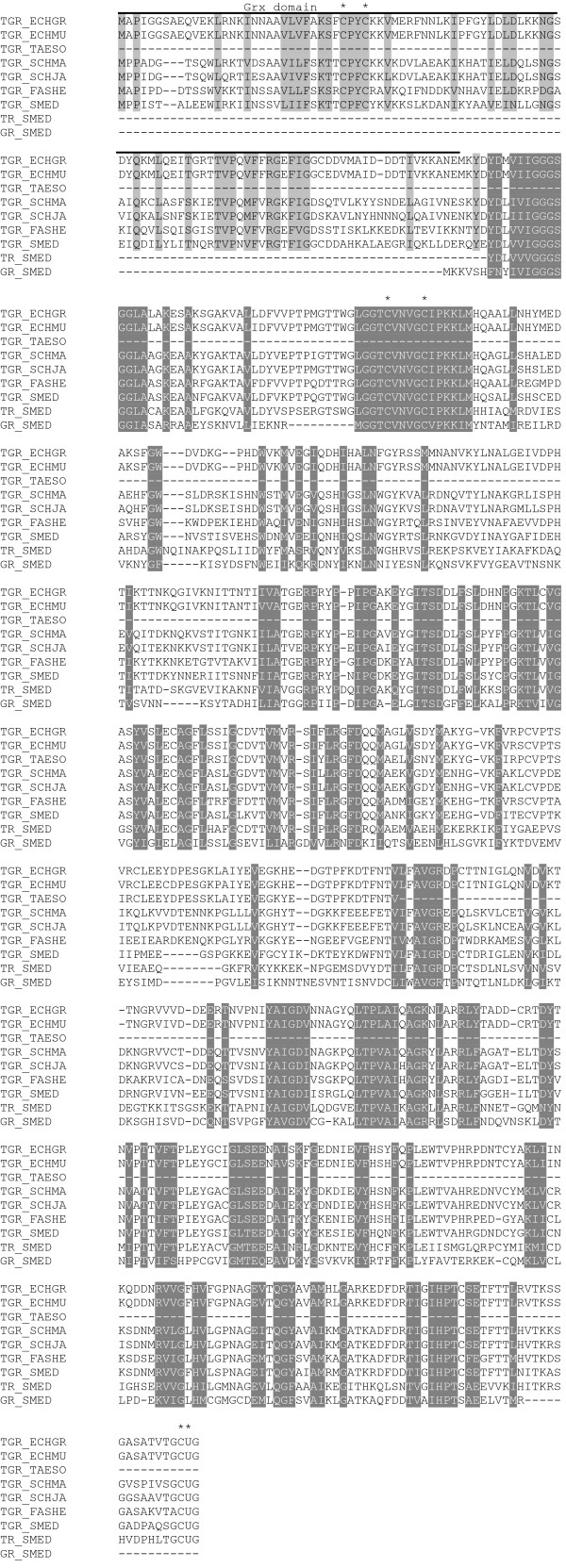
**Amino acid sequence alignment of TR, GR and TGRs of platyhelminths**. Sec is indicated by U. The position of the redox active residues in the sequences is indicated by a star. Conserved residues in all proteins are highlighted in dark grey, conserved residues in the Grx domain of TGRs are highlighted in light grey. Location of the Grx domain is indicated above the sequence. ORFs for TGR, TR and GR from *S. mediterranea *(SCHME) genome (assembly 31) were predicted in the contigs 000676, 000203 and 001663, respectively. ORF for *E. multilocularis *(ECHMU) TGR was assembled from contigs 0007357 and 0007358. Full-length TGR sequences of *S. mansoni *(SCHMA),*S. japonicum *(SCHJA), *E. granulosus *(ECHGR), and *F. hepatica *(FASHE) were retrieved from Genebank (gb|AAK85233.1|AF395822_1, gb|AAW25951.1, emb|CAM96615.1, and gb|AAN63052.1, respectively). *T. solium *(TAESO) TGR partial sequences were retrieved from the EST repository at Genebank (gb|EL757065.1 and gb|EL743442.1). The putative mitochondrial leader peptide of *S. mediterranea *TGR is not included in the sequence, neither leader peptide variants of *Schistosoma *and *Echinococcus *TGRs. Sequences were aligned with Clustal W2 [29], with final manual adjustment after inspection.

We carried out an exhaustive *in silico *analysis of available genome and transcriptome data from platyhelminth organisms to examine the presence of TGR, TR and GR sequences. A tblastn search of the *E. multilocularis *genome using *E. granulosus *TGR sequence as protein query revealed that *E. multilocularis *genome possesses a single TGR gene and lacks genes encoding conventional TR or GR, consistent with previous experimental evidence from *E. granulosus*. *E. multilocularis *TGR is a selenoprotein: its gene contains an in-frame TGA codon as well as a SECIS element. *Echinococcus *TGR orthologs are virtually identical; they possess 95% identity at the nucleotide level and 98% identity at the amino acid level (Figure 1). On the other hand tblastn searches of the *S. mediterranea *genome revealed that, in contrast to the parasitic flatworms *E. multilocularis *(class Cestoda) and *Schistosoma *spp. (class Trematoda), the free-living platyhelminth *S. mediterranea *(class Turbellaria) possesses a TR gene, a GR gene and a TGR gene. The coding sequences of *S. mediterranea *TR, GR and TGR were predicted based on EST sequences when available; final adjustments of intron-exon boundaries were performed based on a multiple alignment of TRs, GRs and TGRs from different species. The deduced amino acid sequences are shown in Figure 1. All *S. mediterranea *pyridine-nucleotide thiol-disulfide oxidoreductases identified contain the canonical CX_4_C redox center. Both TR and TGR genes from *S. mediterranea *contain an in-frame TGA codon and a SECIS element, and thus encode selenoproteins, with a GCUG (U denotes selenocysteine) C-terminal redox center. *S. mediterranea *TGR possesses a dithiol Grx domain, containing the CPFC redox active center, similar to *S. japonicum *TGR and TGRs from other organisms. The TR gene from *S. mediterranea *has higher identity to mammalian mitochondrial TRs. Indeed, an exon encoding a putative mitochondrial leader peptide was detected upstream of the TR coding sequence. In the absence of additional platyhelminth genomes sequenced, we searched databases for expressed TRs, GRs and TGRs of other flatworms. A tblastn search identified a cDNA encoding a Sec-containing full-length TGR from the platyhelminth parasite *Fasciola hepatica *(class Trematoda), with high similarity to *Schistosoma *TGRs and containing the canonical CPYC redox center at the Grx domain, the CX_4_C redox center of pyridine-nucleotide thiol-disulfide oxidoreductases, and a Sec-containing C-terminal redox motif. Additionally, a tblastn search revealed ESTs encoding a fragment of a TGR from the platyhelminth parasite *Taenia solium *(class Cestoda). This TGR is highly similar to *Echinococcus *TGRs. Figure 1 shows an alignment of flatworm TGRs, and *S. mediterranea *TR and GR.

**Figure 2:**
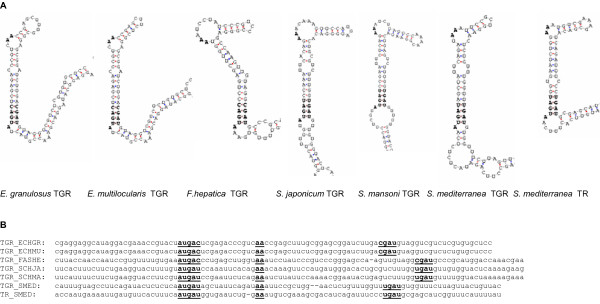
**Structures and nucleotide sequence alignment of SECIS elements of TR and TGRs of platyhelminths**. The SECIS elements were predicted using the SECISearch program [27]. Functionally important nucleotides in the apical loop and the quartet (SECIS core) are shown in bold in the structure and in bold and underlined in the alignment. ECHGR: *E. granulosus*, ECHMU: *E. multilocularis*, SCHMA: *S. mansoni*, SCHJA: *S. japonicum*, SMED: *S. mediterranea*, FASHE: *F. hepatica*.

The SECIS elements of flatworm TGRs and TR are shown in Figure 2. All SECIS structures fit very well the eukaryotic SECIS consensus model, containing a non-Watson-Crick quartet in the SECIS core and unpaired AA in the apical loop. Beyond these regions, little sequence conservation was detected between flatworm TGR SECIS elements.

**Figure 3:**
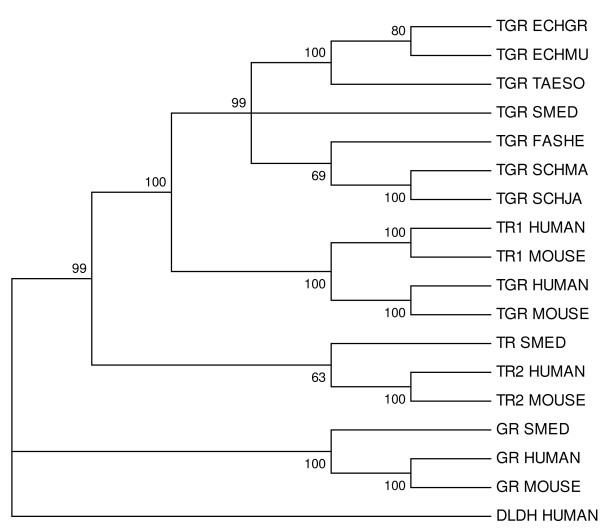
**Phylogenetic relationships of GRs, TRs and TGRs of platyhelminths and mammals**. TRs, GRs and TGRs from platyhelminths and mammals were aligned with Clustal W2 [29]. Human dihydrolipoamide dehydrogenase (DLDH) was used as outgroup. A Neighbor-Joining tree was constructed using MEGA4 [30] with pairwise deletion and default parameters. A condensed tree is shown, and bootstrap values of reliable nodes (above 50) are indicated. The polytomy displayed at the TGR node denotes that the evolutionary relationships within the node can not be resolved with at least 50% of bootstrap support. In other words, nodes with less than 50% of bootstrap support were collapsed and are displayed as polytomies. The results indicate that the TR and GR genes present in the planarian lineage were lost in the neodermata lineage. Very similar topology and statistical support were obtained using different phylogenetic reconstruction methods (*i.e*. Maximum Parsimony, UPMGA and Minimum Evolution). ECHGR: *E. granulosus*, ECHMU: *E. multilocularis*, TAESO: *T. Solium*, SCHMA: *S. mansoni*, SCHJA: *S. japonicum*, SMED: *S. mediterranea*, FASHE: *F. hepatica*.

Phylogenetic analysis of TRs, GRs and TGRs showed that platyhelminth TGRs conform a clade. We could not determine whether platyhelminth TGR is more related to mammalian TR1 (also known as cytosolic TR) or mammalian TGR (Figure 3). *S. mediterranea *TR and GR clustered with mammalian mitochondrial TRs and GRs, respectively. Overall, these results indicate that thioredoxin and glutathione pathways differ in flatworms, and suggest that the TR and GR genes present in the planarian lineage were lost in the neodermata lineage (Figure 3). Finally, a distant paralog of TGR, corresponding to dihydrolipoamide dehydrogenase, was identified in all flatworm genomes.

### Analysis of platyhelminth TGR gene structure

To gain further insights on the structure and evolution of platyhelminth TGRs, we determined the sequence of *E. granulosus *gene and analyzed it together with the sequences of TGRs, TRs and GRs available from flatworm genome projects (*E. multilocularis*, *S. mansoni, S. japonicum *and *S. mediterranea*). The comparison of the gene structure revealed high conservation between parasitic flatworm TGR genes. Indeed, *Schistosoma *spp. and *Echinococcus *spp. TGR genes contained 17 exons of similar sizes for the four species; most exons were small and the longest was the last exon that contained the 3' UTR, including the SECIS element (Table 1). Interestingly, introns were also very well conserved between *Echinococcus *and *Schistosoma *in the glutaredoxin domain; in the TR domains, introns were significantly longer in *Schistosoma *spp. A closer inspection revealed that *E. granulosus *and *E. multilocularis *genes were virtually identical, with the single significant difference of 175 nucleotide insertion at intron 15 (see Table 1), whereas *S. mansoni *and *S. japonicum *sequences differed more markedly in the intron sizes, in particular in the TR domain.

**Table 1:** Exon and intron structure of flatworm TR, GR and TGRs

Exon/Intron	Em_TGR	Eg_TGR	Sma_TGR	Sja_TGR	Sme_TGR	Sme_TR	Sme_GR
							

**E1**	**69**	**69**	**93**	**93**			

I1	281	284	1507	971			

**E2**	**120**	**120**	**108**	**108**	**105**	**55**	**132**

I2	40	40	34	35	45	48	43

**E3**	**61**	**63**	**61**	**61**	**63**	**57**	**116**

I3	41	41	33	30	3066	48	46

**E4**	**87**	**85**	**87**	**87**	**81**	**164**	**70**

I4	118	127	36	36	301	397	44

**E5**	**122**	**122**	**125**	**125**	**132**	**54**	**133**

I5	701	705	2298	2614	2389	52	4105

**E6**	**142**	**142**	**142**	**142**	**144**	**139**	**55**

I6	162	164	214	115	41	9	4262

**E7**	**48**	**48**	**54**	**54**	**48**	**54**	**73**

I7	74	74	2166	179	3006	533	71

**E8**	**143**	**143**	**143**	**143**	**141**	**74**	**246**

I8	300	305	840	643	49	44	8817

**E9**	**116**	**116**	**116**	**116**	**342**	**20**	**378**

I9	121	119	4231	848	998	75	60

**E10**	**226**	**226**	**226**	**226**	**165**	**92**	**216**

I10	885	912	819	1240	59	1704	

**E11**	**105**	**105**	**105**	**105**	**159**	**178**	

I11	211	223	1865	700	58	46	

**E12**	**74**	**74**	**74**	**74**	**339**	**89**	

I12	68	68	2924	2180	1382	18	

**E13**	**154**	**154**	**157**	**157**	**338**	**45**	

I13	198	202	1788	2054		54	

**E14**	**108**	**108**	**108**	**112**		**195**	

I14	81	81	1618	1191		2858	

**E15**	**93**	**93**	**93**	**89**		**63**	

I15	502	678	2491	293		58	

**E16**	**138**	**138**	**138**	**138**		**104**	

I16	305	309	170	908		44	

**E17**	**484**	**485**	**480**	**423**		**378**	

Σtot	6378	6623	25344	16290	13451	7749	18867

**Σexons**	**2290**	**2291**	**2310**	**2253**	**2057**	**1761**	**1419**

The length of exons and introns is shown for *E. multilocularis *TGR (EmTGR), *E. granulosus *TGR (EgTGR), *S. mansoni *TGR (SmaTGR), *S. japonicum *TGR (SjaTGR), *S. mediterranea *TGR (SmeTGR), *S. mediterranea *TR (SmeTR) and *S. mediterranea *GR (SmeGR). Numbering corresponds to *Echinococcus *TGR.

The gene structure of *S. mediterranea *TGR was similar to those of *Echinococcus *and *Schistosoma *TGRs, but had clear differences as well. Indeed, *S. mediterranea *TGR possessed 12 exons, instead of 17. We could not detect the exon containing the signal peptide in the *S. mediterranea *TGR gene, although its presence could not be ruled out, since signal peptide sequences are often poorly conserved. Thus, four events of exon fusion/split appeared to have occurred between neodermata and turbellaria lineages (see Table 1). Interestingly, the exon fusion/split events were not equally distributed in the TGR gene sequence; they occurred in the coding sequence corresponding to the C-terminal half of TGR. Thus, the gene structure appeared to have more constraints at the Grx domain than at the interface domain. In turn, *S. mediterranea *TR and GR genes displayed a completely different exon/intron structure than platyhelminth TGR genes.

Finally, since only a handful of genes from *Echinococcus *have been sequenced, we analyzed in detail various aspects of *Echinococcus *TGR genes. We examined exon-intron boundaries to identify common features and differences (Additional file 1 shows all the exon-intron boundaries of TGR gene). All but one intron contained the canonical GU-AG donor-acceptor sites, typical of eukaryotic nuclear pre-mRNA, being the donor site of intron 15 the exception to the consensus. In addition, we searched for conserved bases or motifs around the splice sites and found that C or A followed by a purine (A or G) preceded the 5' GU splice site in most cases; and that U or A was present at the -3 base with respect to the 3'AG splice site (Additional file 1). The T+A content in TGR introns was 59% (contrasting 50.7% in exons), indicating a neutral mutational bias towards T+A, as previously noted for *Echinococcus *genes [16]. The small size of all *Echinococcus *TGR introns is noteworthy: while *Echinococcus *TGR genes span 6 kb, *S. mediterranea *and *Schistosoma *spp genes are significantly longer. These data agree with the previous observations that *Echinococcus *genes possess small introns [16]. Taken as a whole, our results are also in agreement with previous findings that both *Echinococcus *species are remarkably similar with regard to sequence information.

### Identification of TGR variants in platyhelminths

**Figure 4:**
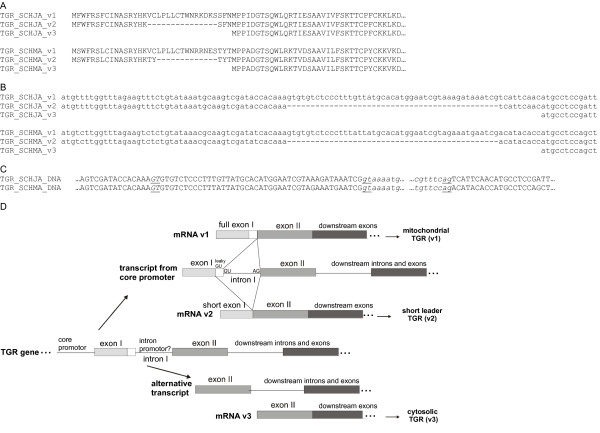
**TGR variants in *Schistosoma *spp**. Amino acid sequence alignment of *S. japonicum *(denoted as SCHJA) and *S. mansoni *(denoted as SCHMA) TGR variants. Variant 1 (v1) encodes a TGR with a mitochondrial signal peptide, variant 2 (v2) encodes a TGR with shorter leader peptide with no topology prediction, and variant 3 (v3) encodes a cytosolic TGR. **B**. Nucleotide sequence alignment of ESTs encoding *S. japonicum *and *S. mansoni *TGR variants; the sequence of SCHMA_v1 was deduced from the corresponding TGR gene. **C**. Nucleotide genomic sequence of *S. mansoni *and *S. japonicum *TGRs. The sequence corresponds to the end of the first exon, the first intron (indicated by italics) and the beginning of the second exon. GT and AG donor and acceptor splice sites of intron I, whose splicing generates variant 1, are shown underlined in lower case. Underlined in capital letters and in italics is shown a presumptive leaky GT donor splice site present in exon I, that, if spliced, gives rise to variant 2. Sequences of variant 3 were retrieved from translated full-cDNAs deposited in Genebank (accession Numbers gb|AAK85233.1|AF395822_1, gb|AAW25951.1), sequences of variants 1 and 2 correspond to ESTs deposited in Genebank (gb|BU801474.1 for Sja variant 1, gb|BU791993.1 and gb|CV688441.1 for Sja2, and gb|CD202891.1 for Sma variant 2). **D**. Proposed model of how *Schistosoma *mRNA variants would be generated. From TGR gene, two primary transcripts would be synthesized from alternative transcription initiation sites: core promoter and a putative intron I promoter. The transcript derived form the core promoter would give rise to two different mRNAs by alternative transcript processing.

We have previously demonstrated the existence of mitochondrial and cytosolic TGR variants derived from a single gene in *E. granulosus*. In order to identify TGR variants expressed in other flatworms, we performed a tblastn search at the NCBI server (EST others option). Only a single TGR variant was identified in the case of *S. mediterranea*. In the case of *S. japonicum*, ESTs encoding two additional TGR variants to the already reported cytosolic TGR were identified, whereas a single additional variant was identified in *S. mansoni*. Similarly to what has been described in *E. granulosus*, the *Schistosoma *cDNAs encode variants differing in their N-termini (Figure 4). One of the *S. japonicum *variants (TGR_SCHJA_v1) would encode an N-terminal mitochondrial signal peptide. The other *S. japonicum *variant (TGR_SCHJA_v2) and the *S. mansoni *variant identified (TGR_SCHMA_v2) encode a leader peptide related to, but shorter than, the mitochondrial one. It is not possible, *in silico*, to ascribe a topological signal to this variant. The genomic sequence and exon-intron structure of *Schistosoma *TGR genes strongly support the existence of these variants. Indeed, the information for the identified *Schistosoma *leader peptides is found in an exon upstream of the one encoding the N-terminal sequence of the Grx domain. TGR_SCHJA_v1 (putatively encoding a mitochondrial TGR variant) and TGR_SCHJA_v2 (with a shorter leader peptide) are derived from alternative splicing of exon I and exon II at a canonical GU donor site present in intron I and at a leaky GU donor site present in exon I, respectively. If the transcript is spliced at this latter GU donor site, it would give rise to a shorter exon I and, consequently to a shorter leader peptide (Figure 4c). The same gene structure is observed in *S. mansoni *TGR suggesting that, in addition to TGR_SCHMA_v2, a mitochondrial variant of TGR (TGR_SCHMA_v1) also exists in this species (Figure 4c). The sequences of the full-length cDNA variants encoding *Schistosoma *cytosolic TGRs, and the structure of the genes suggest that the cytosolic variants (TGR_SCHJA_v3 and TGR_SCHMA_v3) are derived from alternative initiation of transcription, from a putative promoter at intron I, similar to what has been hypothesized for the *E. granulosus *variants. Figure 4d shows a model for the generation of *Schistosoma *TGR variants, which takes into account all this information.

**Figure 5:**
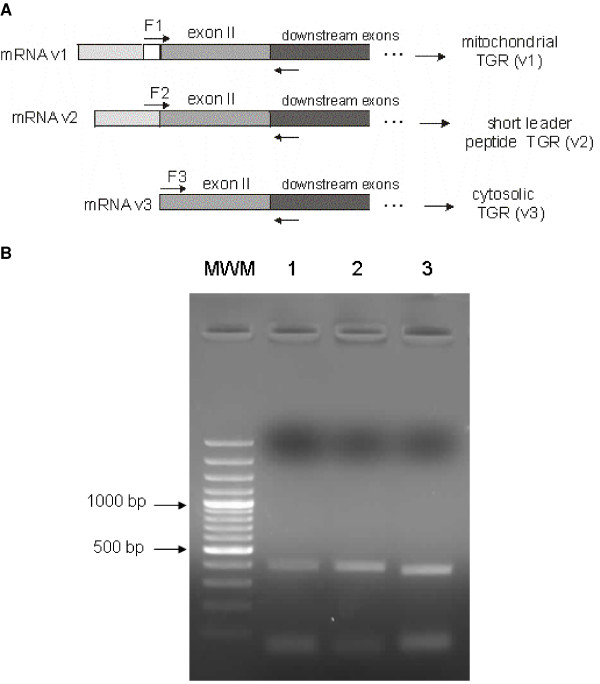
**Expression of *S. mansoni *variants detected by RT-PCR**. **A**. Schematic representation of the three mRNA variants of *S. mansoni *TGR, the variant 1 and variant 2 specific primers (F1 and F2) and the primer corresponding to the 5'-end of the cytosolic variant (F3) used in RT-PCRs in combination with a reverse primer derived from exon 3 sequence. **B**. Electrophoresis of PCR products from *S. mansoni *cercariae: a band of the expected size was observed in the PCRs with F1, F2 and F3 primers and the reverse primer (lanes 2 to 4, respectively). MWM lane corresponds to 100 bp plus ladder (Fermentas).

In order to confirm the presence of the newly identified variant in *S. mansoni *and assess the occurrence of the putative mitochondrial variant (identified in *S. japonicum *ESTs, but not in *S. mansoni*), we performed PCRs from different *Schistosoma *samples (schistosomula, adult worm and cercariae) with forward primers specific for each of the leader peptide variants and a common TGR reverse primer; in addition we used, as a control, a forward primer corresponding to the 5'end of the cytosolic variant (Figure 5a). PCR products of the expected sizes were obtained in the three reactions in all materials, confirming the expression of both leader peptide variants, TGR_SCHMA_v1 and TGR_SCHMA_v2. Figure 5b shows the result of the PCR reactions using cercariae as a cDNA template.

Finally, we investigated whether there are additional variants in *E. granulosus *or whether the already known variants are developmentally regulated. To this end, we performed RT-PCRs using the splice leader exon of *E. granulosus *and TGR reverse specific primers on different *E. granulosus *materials (larval worms, adult worms and germinal layer of hydatid cyst). To allow discrimination of variants that may subtly differ in size, the PCR products obtained were analyzed on polyacrylamide gels. The results suggest that no additional variants to the already described are expressed in *E. granulosus *and that the mitochondrial and cytosolic variants are expressed in all stages analyzed (data not shown).

### Analysis of transcription from alternative promoter site

We previously postulated that the mRNA variants derived from a single TGR gene may arise from two alternative transcription initiation sites located upstream of the first exon (core promoter) and upstream of the second exon (intron promoter), because: i) both variants contained the trans-splice leader exon and ii) a putative TATA box was present within the intron sequence [11]. The comparison of the core promoter and the putative intron promoter nucleotide sequences of *Echinococcus *and *Schistosoma *TGRs did not evidence significant similarity, although there were some conserved regions at both presumptive promoter regions (data not shown). We tested the promoter activity of the putative intron promoter of *E. granulosus *TGR in a heterologous system (CHO-K1 cells) using a reporter vector (pAcGFP1-1™, Clontech), but this approach was not successful. Thus, in order to obtain evidence of alternative transcription initiation in *E granulosus*, we performed RT-PCR with forward primers mapping the first intron and a reverse primer from a downstream exon. This strategy has been previously used [17] and its rationale relies on the fact that trans-splicing is, kinetically, a much slower process than cis-splicing, and thus it is possible to find a population of transcripts not completely processed; these transcripts will have been already cis-spliced, but have not been yet trans-spliced. The results indicate that alternative transcription initiation takes place from intron 1, downstream of the TATA box, since a PCR product was obtained with the forward primer 50 bp downstream of the putative TATA sequence present in the intron, but not with primers immediately downstream or upstream from the TATA sequence. Since TGR mRNAs from *Schistosoma *TGRs are not trans-spliced [13] this strategy could not be used to assess alternative initiation of transcription from intron I.

## Discussion

Previous studies on thioredoxin and glutathione systems in parasitic flatworms indicated that these systems are different to those of their vertebrate hosts [7-9,11]. The main difference is the absence of conventional GR and TR in these parasites, and the substitution of both enzymes by TGR as a sole redox "wire". Our analysis of platyhelminth genomes and transcriptomes indicates that this is the case for all flatworm parasites for which information is available. The *E. multilocularis *genome revealed that this cestode also lacks conventional TR and GR, and its redox thiol-disulfide homeostasis relies exclusively on a single Sec-containing TGR, in agreement with previous biochemical and molecular studies of *E. granulosus*. A cDNA encoding TGR was also identified from *T. solium*, the other medically important cestode. A similar scenario was observed in the case of trematodes: lack of conventional GR and TR and presence of TGR in the genomes of *Schistosoma *spp. [18,19], and a cDNA encoding TGR from *F. hepatica*, an additional member of the class. Thus the dependence on the single oxidoreductase is common to the neodermata lineage (a platyhelminth subphylum that contains the classes Cestoda and Trematoda, but not the free-living class Turbellaria). Our results further revealed that the absence of TR and GR is not characteristic of the entire platyhelminth phylum. The turbellarian *S. mediterranea *possesses TR, GR and TGR, indicating that flatworm parasites have lost TR and GR genes. Elimination of GR and TR from their genomes suggests streamlining of redox regulation.

Another interesting finding in our study is the identification of novel variants of *S. mansoni *and *S. japonicum *TGRs. Previously, cytosolic and mitochondrial variants derived from a single gene were described in *E. granulosus *[11]. In the case of *Schistosoma*, we could detect *in silico *and confirm experimentally the existence of two variants in addition to the previously reported cytosolic form. One of the novel forms encodes a mitochondrial TGR; and this is in agreement with the occurrence of cytosolic and mitochondrial thioredoxins in *Schistosoma *[18]. The second newly identified TGR variant encodes a shorter leader peptide. The functional significance of this leader peptide (*e.g*. topological localization information or a regulatory role) must await functional studies. In many species, distinct genes encode for mitochondrial and cytosolic TRs. Nevertheless, complex expression patterns and rare mRNA variants have also been observed for all TR genes in mammals [20-26], and the functional significance of this complexity is not fully understood. In this context, it is important to note that the three variants of *S. mansoni *TGR and the cytosolic and mitochondrial variants of *E. granulosus *are expressed in all the life cycle stages examined (cercariae, schistosomule and adult worms in the case of *S. mansoni*; larval worms, hydatid cyst wall and adult worms in the case of *E. granulosus*). The existence of TGR variants reveals a relatively complex expression of the gene in these organisms that may be relevant when considering TGR as a drug target; in particular if inhibition is to be achieved at different subcellular compartments.

The study of the mechanism by which TGR variants may be generated in parasitic flatworms was addressed by two different strategies. The initial promoter reporter strategy was not successful. However, in the case of *E. granulosus *it was possible to map the initiation of transcription from the intron promoter, downstream from the presumptive TATA box. Thus, our results suggest that two promoters (core and intron promoters) give rise to two different transcripts. The absence of the splice leader exon in *Schistosoma *TGR mRNAs precluded the use of this strategy. Nevertheless, the information is consistent with the model depicted in Figure 4d. One of the transcripts would be derived from the core promoter and would be alternatively spliced, generating the two variants with related but different N-terminal signal peptides, and an additional variant would be generated from alternative initiation of transcription.

## Conclusions

Our study found that the biochemical scenario in parasitic flatworms differs from the one observed in free-living flatworms. A unique and simplified redox system, termed linked thioredoxin-glutathione system, is present in all platyhelminth parasites examined. In contrast, conventional and linked thioredoxin and glutathione systems were found in *S. mediterranea*, a free-living platyhelminth. The data also show that canonical GR and TR were specifically lost in the parasitic platyhelminths. Overall, our results reinforce previous studies indicating that TGR is an excellent drug target for flatworm infections [14,15]; indeed, maintenance of redox homeostasis and basic cellular processes such as synthesis of deoxyribonucleotides depend on a single selenoenzyme in different classes of flatworm parasites. Furthermore, the phylogenetic analysis of TRs, GRs and TGRs indicates that flatworm TGRs form a clade with no clear ortholog to either mammalian TRs or TGR; these differences may be relevant for selective inhibition. The fact that all flatworm TGRs contain selenocysteine at the active site constitutes a unique opportunity to target this particularly reactive nucleophilic residue. Alternatively, flatworm parasite redox homeostasis could be disrupted targeting selenium incorporation, for instance by selectively inhibiting platyhelminth selenophosphate synthetase or SECIS-binding protein.

Our work also demonstrates the expression of TGR variants in the human parasites *Schistosoma *spp. Finally, we provide evidence that alternative initiation of transcription and transcript processing contribute to the generation of TGR variants in platyhelminth parasites, revealing plasticity of gene expression in these organisms.

## Methods

### Identification of TR, GR and TGR genomic, cDNA and coding sequences in platyhelminth databases

In order to obtain information on platyhelminth TR, GR and TGR sequences, the *S. mansoni*, *S. japonicum, E. multilocularis *and *S. mediterranea *genomic databases (http://www.sanger.ac.uk/Projects/S_mansoni, http://lifecenter.sgst.cn/schistosoma/en/schistosomeBlast.do, http://www.sanger.ac.uk/Projects/Echinococcus, and http://smedgd.neuro.utah.edu, respectively) were searched with tblastn, using the sequences of *S. mansoni *and *E. granulosus *TGRs and human GR, TRs and TGR as queries. The coding sequence of *E. multilocularis *TGR was determined based on the cDNA and amino acid sequences of *E. granulosus *TGR previously described in [11]. To map the coding sequence of *S. mediterranea *TGR, TR and GR, the amino acid sequences of *E. granulosus *and *S. mansoni *TGRs, human mitochondrial TR and human GR, were respectively used, as well as available ESTs encoding *S. mediterranea *TGR, TR and GR, retrieved from http://smedgd.neuro.utah.edu/. The final adjustments of intron-exon boundaries were performed based on a multiple alignment of TRs, GRs and TGRs from different species. Finally, in order to obtain information on expressed TR, GR and TGR from other platyhelminth organisms, tblastn (EST others option) and blastp searches were performed at the NCBI server with flatworm and mammalian pyridine-nucleotide thiol-disulfide oxidoreductases as queries.

### Sequence and phylogenetic analyses

The selenocysteine insertion sequence (SECIS) elements present in flatworm TR and TGR genes were identified using the SECISearch program, version 2-19 http://genome.unl.edu/SECISearch.html[27] using the default energy cutoffs in all cases, and the canonical pattern for all but *Schistosoma *TGRs; in these latter instances the non-canonical pattern revealed the SECIS element. Topology prediction of the polypeptides was carried out using SignalP 3.0 [23] and ipSORT [28]. Phylogenetic analyses of flatworm and mammalian GRs, TRs and TGRs were performed on amino acid sequences aligned with ClustalW2 [29]. Neighbor-joining, maximum parsimony, minimum evolution and UPMGA methods were used to reconstruct phylogenies, with MEGA4 [30], and the following parameters: pairwise deletion, Poisson correction and uniform rates among sites. In all cases bootstrap test was performed on 500 replicates. The analyses were performed including and excluding the Grx domain of TGR.

### In silico and experimental identification of platyhelminth TGR variants

To identify platyhelminth TGR variants, tblastn searches were performed on the blast servers of NCBI (option EST others) and *S. mansoni*, *E. multilocularis *and *S. mediterranea *genomic databases using the TGR sequences as queries. Experimental evidence of newly identified *S. mansoni *variants was analyzed using forward primers that allow discrimination of the variants (for details see Figure 5) used in combination with a TGR gene-specific reverse primer in PCR reactions from cDNAs of different *S. mansoni *materials. Total RNA was isolated from cercariae, 3 hours schistosomula and adult worms using a modified TRIZOL (Invitrogen)/RNeasy (Qiagen) procedure [31]. Schistosomula were prepared by mechanical transformation of cercariae as previously described [32]. cDNA was generated from 1 μg of total RNA using Superscript II (Invitrogen) according to manufacturer's specifications. The *S. mansoni *life cycle is maintained at the Schistosoma Research Group at the Pathology Department, University of Cambridge, UK. In the case of *E. granulosus*, experimental identification of additional TGR variants was examined on different parasite materials (larval worms, adult worms and hydatid cyst wall) by RT-PCR, using *E. granulosus *5' splice-leader exon as forward primer and different gene specific reverse primers derived from the previously published TGR cDNA sequence [11]. In all cases RNA was obtained from trizol-treated *E. granulosus *samples and subsequently used as template for reverse transcription reactions and PCRs as previously described [33], using ThermoScript (Invitrogen) reverse transcriptase and Pfu (Fermentas) DNA polymerase, respectively. In all cases, PCR products were analyzed by electrophoresis.

### Amplification of Echinococcus granulosus TGR gene and its promoter from genomic DNA

Two fragments corresponding to the Grx domain and the TR domains of the TGR gene were amplified by PCR from *E. granulosus *DNA, using forward and reverse primers derived from *E. granulosus *TGR cDNA sequence. The PCR products were cloned in pGEM-T easy™ (Promega) and subsequently sequenced by primer walking. The core promoter of TGR gene (1.5 kbp sequence upstream of TGR coding sequencer) was amplified using a forward primer derived from *E. multilocularis *homologous region, and a reverse primer derived from the 5'-end of *E. granulosus *TGR cDNA sequence; this PCR product was cloned and sequenced as described above.

### Analysis of alternative initiation of transcription from *E. granulosus *TGR gene

**Figure 6:**
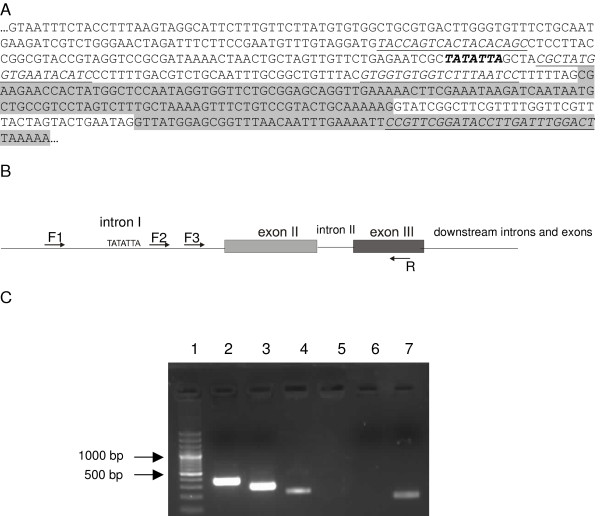
**Analysis of alternative initiation of transcription from *E. granulosus *TGR intron 1**. **A**. The sequence of *E. granulosus *TGR gene starting from intron 1 ending at exon 3. Exons 2 and 3 are shaded in grey. The putative TATA box present in intron 1 is highlighted in bold and italics. Sequences of the primers used in the PCR experiments are shown underlined and in italics: forward primers 1, 2 and 3 span intron 1 from 5' to 3'; the reverse primer derives from exon 3 sequence. **B**. Schematic representation of the primers used in PCR reactions, localized in the TGR sequence. **C**. Gel-electrophoresis of PCR reaction carried out from genomic DNA (lanes 2 to 4) and from *E. granulosus *cDNA (lanes 5 to 7) using a combination of the reverse primer and forward primers 1, 2 and 3 (lanes 2 and 5, lanes 3 and 6, lanes 4 and 7, respectively). Lane 1 corresponds to 100 bp plus ladder (Fermentas).

In order to investigate and map alternative transcription initiation, we also performed RT-PCRs from *E. granulosus *hydatid cyst germinal layer using forward primers spanning the first intron of *E. granulosus *TGR (upstream and downstream of the presumed TATA box present in intron 1) and a reverse primer corresponding to exon 3 (Figure 6). Control PCRs were carried out from genomic DNA.

## Abbreviations

GSH: Glutathione; GR: glutathione reductase; GSSG: oxidized glutathione; SECIS; selenocysteine insertion sequence; Trx: thioredoxin; TGR: thioredoxin glutathione reductase; TR: thioredoxin reductase; EST: expressed sequence tag.

## Authors' contributions

LO carried out most of the work described: cloned *E. granulosus *gene and promoter, performed the experiments using a heterologous system, analyzed the existence of TGR variants in *S. mansoni *and *E. granulosus *cDNAs by PCR, searched the genomes of *E. multilocularis*, *Schistosoma *spp. and *S. mediterranea *and analyzed the gene structure of the genes of interest. AVP prepared mRNA and cDNA from different *S. mansoni *samples. CF and MB prepared cDNA and genomic DNA from *E. granulosus *samples and provided technical help. GS performed *in silico *data mining of ESTs and genomes, phylogenetics analysis, and performed the experiments to test alternative transcription from intron promoter by PCR. LO and GS primarily conceived and designed the study, with insights from CF and VNG. Analyzed all the data: LO, VNG, CF and GS. GS wrote the manuscript, LO, VNG and CF helped to draft the manuscript. All authors read, revised and approved the final manuscript.

## Supplementary Material

Additional file 1 ***Echinococcus granulosus *and *Echinococcus multilocularis *TGR exon-intron boundaries**. Exon-intron boundary sequences of TGRs and canonical donor and acceptor splice sites. Click here for file

## References

[B1] Littlewood DTJ Maule AG, Marks NJ The Evolution of Parasitism in Flatworms Parasitic flatworms: molecular biology, biochemistry, immunology and physiology 1 CAB International 1 36

[B2] Hotez PJ Brindley PJ Bethony JM King CH Pearce EJ Jacobson J Helminth infections: the great neglected tropical diseases J Clin Invest 2008 118 4 1311 1321 10.1172/JCI34261 18382743 PMC2276811

[B3] Garcia HH Moro PL Schantz PM Zoonotic helminth infections of humans: echinococcosis, cysticercosis and fascioliasis Curr Opin Infect Dis 2007 20 5 489 494 10.1097/QCO.0b013e3282a95e39 17762782

[B4] Cioli D Valle C Angelucci F Miele AE Will new antischistosomal drugs finally emerge? Trends Parasitol 2008 24 9 379 382 10.1016/j.pt.2008.05.006 18675590

[B5] Toledano MB Kumar C Le Moan N Spector D Tacnet F The system biology of thiol redox system in Escherichia coli and yeast: differential functions in oxidative stress, iron metabolism and DNA synthesis FEBS Lett 2007 581 19 3598 3607 10.1016/j.febslet.2007.07.002 17659286

[B6] Nordberg J Arner ESJ Reactive oxygen species, antioxidants, and the mammalian thioredoxin system Free Radical Biology and Medicine 2001 31 11 1287 1312 10.1016/S0891-5849(01)00724-9 11728801

[B7] Alger HM Williams DL The disulfide redox system of *Schistosoma mansoni *and the importance of a multifunctional enzyme, thioredoxin glutathione reductase Mol Biochem Parasitol 2002 121 1 129 139 10.1016/S0166-6851(02)00031-2 11985869

[B8] Rendón JL del Arenal IP Guevara-Flores A Uribe A Plancarte A Mendoza-Hernández G Purification, characterization and kinetic properties of the multifunctional thioredoxin-glutathione reductase form *Taenia crassiceps *metacestode (cysticerci) Molecular and Biochemical Parasitology 2004 133 200461 69 10.1016/j.molbiopara.2003.09.003 14668013

[B9] Salinas G Selkirk ME Chalar C Maizels RM Fernández C Linked thioredoxin-glutathione systems in platyhelminths Trends in Parasitology 2004 20 7 340 346 10.1016/j.pt.2004.05.002 15193566

[B10] Sun QA Wu Y Zappacosta F Jeang KT Lee BJ Hatfield DL Gladyshev VN Redox regulation of cell signaling by selenocysteine in mammalian thioredoxin reductases J Biol Chem 1999 274 35 24522 24530 10.1074/jbc.274.35.24522 10455115

[B11] Agorio A Chalar C Cardozo S Salinas G Alternative mRNAS arising from trans-splicing code for mitochondrial and cytosolicvariants of *Echinococcus granulosus *thioredoxin glutathione reductase The Journal of Biological Chemistry 2003 278 15 12920 12928 10.1074/jbc.M209266200 12538593

[B12] Bonilla M Denicola A Novoselov SV Turanov AA Protasio A Izmendi D Gladyshev VN Salinas G Platyhelminth mitochondrial and cytosolic redox homeostasis is controlled by a single thioredoxin glutathione reductase and dependent on selenium and glutathione J Biol Chem 2008 283 26 17898 17907 10.1074/jbc.M710609200 18408002 PMC2440607

[B13] Alger HM Sayed AA Stadecker MJ Williams DL Molecular and enzymatic characterisation of *Schistosoma mansoni *thioredoxin International Journal for Parasitology 2002 32 10 1285 1292 10.1016/S0020-7519(02)00108-X 12204228

[B14] Kuntz AN Davioud-Charvet E Sayed AA Califf LL Dessolin J Arner ES Williams DL Thioredoxin glutathione reductase from *Schistosoma mansoni *: an essential parasite enzyme and a key drug target PLoS Med 2007 4 6 e206 10.1371/journal.pmed.0040206 17579510 PMC1892040

[B15] Lea WA Jadhav A Rai G Sayed AA Cass CL Inglese J Williams DL Austin CP Simeonov A A 1,536-well-based kinetic HTS assay for inhibitors of Schistosoma mansoni thioredoxin glutathione reductase Assay Drug Dev Technol 2008 6 4 551 555 10.1089/adt.2008.149 18665782 PMC2669305

[B16] Fernandez V Zavala A Musto H Evidence for translational selection in codon usage in Echinococcus spp. Parasitology 2001 123 Pt 2 203 209 11510686 10.1017/s0031182001008150

[B17] Brehm K Jensen K Frosch M mRNA trans-splicing in the human parasitic cestode *Echinococcus multilocularis* J Biol Chem 2000 275 49 38311 38318 10.1074/jbc.M006091200 10973970

[B18] Berriman M Haas BJ LoVerde PT Wilson RA Dillon GP Cerqueira GC Mashiyama ST Al-Lazikani B Andrade LF Ashton PD The genome of the blood fluke *Schistosoma mansoni* Nature 2009 460 7253 352 358 10.1038/nature08160 19606141 PMC2756445

[B19] The Schistosoma japonicum Genome Sequencing and Functional Analysis Consortium The Schistosoma japonicum genome reveals features of host-parasite interplay Nature 2009 460 7253 345 351 10.1038/nature08140 19606140 PMC3747554

[B20] Dammeyer P Damdimopoulos AE Nordman T Jimenez A Miranda-Vizuete A Arner ES Induction of cell membrane protrusions by the N-terminal glutaredoxin domain of a rare splice variant of human thioredoxin reductase 1 J Biol Chem 2008 283 5 2814 2821 10.1074/jbc.M708939200 18042542

[B21] Rundlof AK Janard M Miranda-Vizuete A Arner ES Evidence for intriguingly complex transcription of human thioredoxin reductase 1 Free Radic Biol Med 2004 36 5 641 656 10.1016/j.freeradbiomed.2003.12.004 14980707

[B22] Turanov AA Su D Gladyshev VN Characterization of alternative cytosolic forms and cellular targets of mouse mitochondrial thioredoxin reductase J Biol Chem 2006 281 32 22953 22963 10.1074/jbc.M604326200 16774913

[B23] Bendtsen JD Nielsen H von Heijne G Brunak S Improved prediction of signal peptides: SignalP 3.0 J Mol Biol 2004 340 4 783 795 10.1016/j.jmb.2004.05.028 15223320

[B24] Gladyshev VN Liu A Novoselov SV Krysan K Sun QA Kryukov VM Kryukov GV Lou MF Identification and characterization of a new mammalian glutaredoxin (thioltransferase), Grx2 J Biol Chem 2001 276 32 30374 30380 10.1074/jbc.M100020200 11397793

[B25] Damdimopoulos AE Miranda-Vizuete A Treuter E Gustafsson JA Spyrou G An alternative splicing variant of the selenoprotein thioredoxin reductase is a modulator of estrogen signaling J Biol Chem 2004 279 37 38721 38729 10.1074/jbc.M402753200 15199063

[B26] Matsuzaka Y Okamoto K Mabuchi T Iizuka M Ozawa A Oka A Tamiya G Kulski JK Inoko H Identification and characterization of novel variants of the thioredoxin reductase 3 new transcript 1 TXNRD3NT1 Mamm Genome 2005 16 1 41 49 10.1007/s00335-004-2416-y 15674732

[B27] Kryukov GV Castellano S Novoselov SV Lobanov AV Zehtab O Guigo R Gladyshev VN Characterization of Mammalian Selenoproteomes Science 2003 300 5624 1439 1443 10.1126/science.1083516 12775843

[B28] Bannai H Tamada Y Maruyama O Nakai K Miyano S Extensive feature detection of N-terminal protein sorting signals Bioinformatics 2002 18 2 298 305 10.1093/bioinformatics/18.2.298 11847077

[B29] Larkin MA Blackshields G Brown NP Chenna R McGettigan PA McWilliam H Valentin F Wallace IM Wilm A Lopez R Clustal W and Clustal X version 2.0 Bioinformatics 2007 23 21 2947 2948 10.1093/bioinformatics/btm404 17846036

[B30] Tamura K Dudley J Nei M Kumar S MEGA4: Molecular Evolutionary Genetics Analysis (MEGA) software version 4.0 Mol Biol Evol 2007 24 8 1596 1599 10.1093/molbev/msm092 17488738

[B31] Hoffmann KF Johnston DA Dunne DW Identification of *Schistosoma mansoni *gender-associated gene transcripts by cDNA microarray profiling Genome Biol 2002 3 8 RESEARCH0041 10.1186/gb-2002-3-8-research0041 12186648 PMC126235

[B32] Brink LH McLaren DJ Smithers SR *Schistosoma mansoni *: a comparative study of artificially transformed schistosomula and schistosomula recovered after cercarial penetration of isolated skin Parasitology 1977 74 1 73 86 10.1017/S0031182000047545 320543

[B33] Fernandez C Gregory WF Loke P Maizels RM Full-length-enriched cDNA libraries from *Echinococcus granulosus *contain separate populations of oligo-capped and trans-spliced transcripts and a high level of predicted signal peptide sequences Mol Biochem Parasitol 2002 122 2 171 180 10.1016/S0166-6851(02)00098-1 12106871

