# Supplementary material for: Thioredoxin and glutathione systems differ in parasitic and free-living platyhelminths
Source: BMC Genomics. 2010 Apr 13;11:237. doi: 10.1186/1471-2164-11-237 (PMC2873472; doi:10.1186/1471-2164-11-237)
Supplement: Additional file 1 — Echinococcus granulosus and Echinococcus multilocularis TGR exon-intron boundaries. Exon-intron boundary sequences of TGRs and canonical donor and acceptor splice sites. [file 1471-2164-11-237-S1.DOC]

*Echinococcus granulosus* and *Echinococcus multilocularis* TGR exon-intron boundaries.

1Em AACCCGguaauuuc … cuuuuuagCGAAGA

1Eg AAUCCGguaauuuc … cuuuuuagCGAAGA

2Em AAAAAGguaucggc … cugaauagGUUAUG

2Eg AAAAAGguauccgc … cugaauagGUUAUG

3Em UAAAAAgugcgugc … uuuaauagAGAAUG

3Eg AAAAAGgugcgugc … cauuagagAAUGGA

4Em UCAUCGguaagcuu … ugcuccagGUGGUU

4Eg UCAUCGguaagcuu … ugcuccagGUGGUU

5Em GCAAAGgucagcua … accugcagGAGUCG

5Eg GCUAAGgucagcua … accugcagGAAUCG

6Em UCUUAAgugagucu … ucuuacagAUCACU

7Eg UCUUAAgugagucu … ucuuacagAUCACU

8Em AUAAAGguaugauu … uccaauagGUCCUC

8Eg AUAAAGguaugauu … uccaauagGUCCUC

9Em AUCAAGgugauuua … acuuguagACUACA

9Eg AUCAAGgugauuua … acuuguagACUACA

10Em CACUAGguuaguac … cauugcagUGACGA

10Eg CACUAGguuaguac … cauugcagUGACGA

11Em ACUUCUguaaguuc … ccuuucagGUUCGA

11Eg ACUUCUguaaguuc … cuuuucagGUUCGA

12Em AACACGguaagcuc … ugcuauagGUCCUG

12Eg AACACGguaagcuc … cgcuauagGUCCUG

13Em CACCAAgugaguca … uucaaaagUGGCCG

13Eg CACCAAgugaguca … uucaaaagUGGCCG

14Em UGUCGGguaagccc … uuuuucagACGGAU

14Eg UGUCGGguaagccc … uuuuucagACGGAU

15Em AUCGAGgcgaguaa … ccuuuuagGUCUUC

15Eg AUCGAGgcgaguaa … ccuuuuagGUCUUC

16Em CAGGACgugaguuu … cuccacagGACAAC

16Eg CAGGACgugaguuu … cuccacagGACAAC

17Em UCGGAGgugagucg … ccgugaagACUUUC

17Eg UCAGAGgugagucg … ccgugaagACUUUC

Exon sequences are shown in capital letter; grey and turquoise correspond to *E. multilocularis* and *E. granulosus*, respectively. Introns are denoted by lower case; canonical donor and acceptor splice sites (GU and AG, respectively) are highlighted in yellow. A non-canonical donor site present in intron 15 is indicated in fuchsia.
